# Molecular docking analysis of FAS II from *Neisseria meningitides* with marine phytochemicals

**DOI:** 10.6026/973206300223752

**Published:** 2026-06-30

**Authors:** Priyanka Kumari, Diksha Ranga, Pooja Yadav, Vandana Saini, Ajit Kumar

**Affiliations:** 1 Toxicology and Computational Biology Group, Centre for Bioinformatics, Maharshi Dayanand University, Rohtak, Haryana, India, 124001

**Keywords:** Molecular docking, comprehensive marine natural products database (CMNPD), homology modeling

## Abstract

*Neisseria meningitidis* remains a serious global health threat due to rapid progression, high mortality and rising antibiotic resistance. FabF, a key enzyme in the bacterial fatty acid synthesis (FAS II) pathway, is a promising target for new antimicrobials. A 3D model of FabF was constructed and validated and 2,583 marine natural compounds were screened for drug-likeness and ADMET properties. Molecular docking of 241 selected compounds identified three candidates with stronger binding affinity than minocycline. Compounds CMNPD5392, CMNPD14910 and CMNPD14926 showed promising interactions, indicating potential as FabF-targeted therapeutics.

## Background:

Meningitis is an infection characterized by the inflammation of the delicate membranes surrounding the brain and spinal cord. It accounts for nearly 80% of all cases [1] with 5-10% having meningococcus in their nasopharynx asymptomatically [2, 3]. N. *meningitidis* is categorized into 13 serogroups based on their make-up of the polysaccharide capsular antigens [4]. For designing new generation novel antibiotics the key enzymes and their acyl groups' carrier involved in fatty acid synthases (FAS) bears a great potential for serving as target protein [5, 6, 7]. Various effective drugs are available for the treatments of Group B organism's infections. These drugs also have some possible drawbacks like antibiotic resistance developed by bacteria over time which means that some strains of these bacteria are less susceptible to the drugs which can lower the effectiveness of the drugs [8]. Therefore, it is of interest to screen phytochemicals and model the 3D structure of the 3-oxoacyl synthase II protein of N. *meningitides*

## Materials and Methodology:

Our work involved high throughput virtual screening of marine phytochemicals for tweaking FAS II pathway by inhibiting 3-oxoacyl synthase II protein of *N. meningitides* (Figure 1 - see PDF).

## Retrieval of marine phytochemicals:

CMNPD (Comprehensive Marine Natural Products Database) (https://www.cmnpd.org) is a manually curated openly accessible knowledge base database designed for marine natural products research [9].The database includes information on chemical substances with a broad range of physicochemical and pharmacokinetic properties, as well as data on biological activity, systematic taxonomy and topographical distribution of the organisms that make up the database [9]. Its current release CMNPD 1.0 includes 32000 compounds, 3400 organisms, 2700 targets, 72000 bioactivities and 128000 documents.

## Drug likeliness screening:

Retrieved marine phytochemicals were checked for their drug-likeliness properties based on Lipinski's rule of five [1] while other physicochemical data for the retrieved phytochemicals already existed on the CMNPD and were used for drug, these compounds were filtered using the Lipinski parameters indicated above.

## Pharmocokinetic profile screening:

Druggable marine phytochemicals were further screened for pharmacokinetic properties. Pharmacokinetic or ADMET properties are the key features that determine a drug's uptake, elimination, metabolic behaviour, effectiveness and safety [11]. Selected phytochemicals were checked for their absorption (Human intestinal absorption, Blood-brain-barrier permeability and oral bioavailability) and toxicity (hepatotoxicity, Ames's mutagens, carcinogens and toxicophores) using the pkCSM webserver [12]. Compounds showing favourable absorption-related properties and negative scores for toxicity parameters were selected for further studies.

## Phytochemical structure preparation for docking:

BBB permeable and non-toxic drug like marine phytochemicals were selected for docking studies; their 3D structure was retrieved from CMNPD as a single SDF file. These files were separated as a single file and converted as 3D for each marine phytochemical using the open Babel tool. All the 3D structures were dock prep using UCSF-Chimera and were saved as PDF files.

## Target receptor selection:

The three-dimensional structure of N. *Meningitidis* 3-oxoacyl synthase II was not available and was hence generated using homology modeling approach. The sequence of 3-oxoacyl synthase II from N. meningitidis serogroups B (Strain H44/76, ID: E6MWA6) was retrieved from UniProt at https://www.uniprot.org/ (UniProt Consortium - Nucleic acids research, 2015). The protein 3-oxoacyl synthase II sequence was observed to be of 415 amino acids.

## Homology modelling and model evaluation:

The 3D structure model of the required 3-oxoacyl synthase II protein was generated using SWISS-MODEL (https://swissmodel.expasy.org/) [13, 14]. Subsequently, the model was subjected to validation using the SAVES server and ProSa-web [15]. The SAVES server incorporates PROCHECK, ERRAT [16] and VERIFY3D [17] for validation purposes. Once validated, the generated protein structure was subjected to energy minimization using UCSF-Chimera [18].

## Standard drug selection and ligand screening:

Minocycline was chosen as the standard drug based on previous studies related to meningococcus diseases [19, 20]. Minocycline structure was retrieved from PubChem. Based on the ADMET properties of CMNPD compounds, 242 compounds, showing drug-like properties, were selected as ligands for molecular docking studies.

## Molecular docking studies:

Molecular docking was performed using AutoDock 4.2.6 [21]. Initially, both Minocycline and the 3-oxoacyl synthase II protein were optimized and subjected to docking using the AutoDock 4.2.6 software. The ligand and receptor molecule was converted into PDBQT format for the docking process. Ligand compound was docked with receptor protein using default parameters as specified in AutoDock and the binding energy was observed. The docked complexes of these compounds were subsequently analyzed using LigPlot software to investigate the protein-ligand interactions within their complexes, specifically examining hydrogen bonds and hydrophobic contacts.

## Results and Discussion:

A total of 2,583 marine phytochemical compounds were retrieved from the Comprehensive Marine Natural Products Database (CMNPD) along with their associated physicochemical properties. The dataset was compiled into a CSV file containing detailed information on pharmacokinetic attributes, biological activities, systematic taxonomy and the geographical distribution of the source organisms [9]. These marine-derived compounds were initially evaluated for drug-likeness using Lipinski's rule of five. Of the 2,583 phytochemicals screened, 1,217 compounds satisfied the drug-likeness criteria and were advanced for further analysis. Pharmacokinetic and toxicity profiling is essential for predicting a compound's absorption, distribution, metabolism, excretion and safety characteristics [11]. Accordingly, the 1,217 shortlisted phytochemicals were subjected to ADMET screening using the pkCSM web server [12]. Parameters related to absorption (human intestinal absorption, blood-brain barrier permeability and oral bioavailability) and toxicity (hepatotoxicity, Ames mutagenicity, carcinogenicity and toxicophore presence) were assessed. Based on these criteria, 241 marine phytochemicals demonstrated favorable ADMET profiles, including good oral bioavailability,BBB permeability and non-toxic behavior. These compounds were therefore selected for subsequent molecular docking studies (Table 1).The amino acid sequence of 3-oxoacyl-ACP synthase II (FabF) from *Neisseria meningitidis* serogroup B (UniProt ID: E6MWA6) was used to construct a three-dimensional structural model. The crystal structure of Escherichia coli beta-ketoacyl-ACP synthase II (PDB ID: 1KAS) served as the template for homology modeling. Both target and template sequences were submitted in FASTA format to the SWISS-MODEL server, resulting in the generation of a high-quality FabF model (Figure 2 - see PDF). Comprehensive structural validation using standard assessment tools confirmed the reliability and structural integrity of the modeled protein, indicating its suitability for structure-based drug discovery studies (Table 2).A total of 242 marine phytochemicals, including the reference drug minocycline, were docked against the energy-minimized FabF model using AutoDock 4.2.6. Comparative docking analysis revealed that three phytochemicals- CMNPD5392, CMNPD14910 and CMNPD14926 exhibited stronger binding affinities than the standard drug minocycline (Table 3). These bioactive compounds were derived from marine red algae belonging to the genera *Laurencia* and *Osmundea*, which have been widely reported for their diverse pharmacological activities, including antibacterial and neuroprotective effects. Notably, crude extracts of *Osmundeahybrida* have demonstrated antibacterial activity against *Mycobacterium smegmatis* [22], while *Laurenciaokamurae* and other *Laurencia species* have shown inhibitory activity against food-borne pathogens, methicillin-resistant Staphylococcus aureus, Streptococcus pyogenes, Salmonella spp. and Vibrio cholera [23, 24]. Ligand-protein interaction analyses were performed using LigPlot Plus to compare the binding modes of the top-ranked phytochemicals with that of minocycline (Figure 3 - see PDF). The results indicated that CMNPD5392, CMNPD14910 and CMNPD14926 occupied the same active-site pocket as the standard drug. The binding pocket of minocycline consisted of residues Phe401, Thr272, Pro274, Thr307, Pro308, Leu309, Ala273, Met207, Ala209, Lys208 and Gly230, forming hydrogen bonds with Thr272, Ala209, Met207 and Lys208 at bond distances of 2.92, 3.23, 3.34 and 2.71 Å, respectively. The comparable binding interactions observed for the identified phytochemicals further support their potential as promising FabF inhibitors.

## Conclusion:

We report the favorable binding of selected marine-derived phytochemicals to 3-oxoacyl-ACP synthase II from *N. meningitidis*, a key enzyme in the FAS II pathway. The compounds CMNPD5392, CMNPD14910 and CMNPD14926 exhibited stronger binding affinities and occupied the same active-site pocket as the reference drug minocycline. These findings highlight their potential as promising lead candidates for the development of FabF-targeted therapeutics against *N. meningitidis*.

## Figures and Tables

**Table 1:** List of marine phytochemicals of Comprehensive Marine Natural Products Database (CMNPD), selected for molecular docking studies (https://www.cmnpd.org)

**CMNPD10251**	**CMNPD14921**	**CMNPD22370**	**CMNPD31514**	**CMNPD675**
CMNPD14909	CMNPD14926	CMNPD22373	CMNPD31515	CMNPD69
CMNPD14910	CMNPD15655	CMNPD22375	CMNPD31516	CMNPD70
CMNPD14912	CMNPD15850	CMNPD22379	CMNPD31525	CMNPD701
CMNPD14913	CMNPD15851	CMNPD22386	CMNPD31550	CMNPD702
CMNPD10252	CMNPD15852	CMNPD2260	CMNPD3211	CMNPD704
CMNPD10253	CMNPD15856	CMNPD2261	CMNPD3212	CMNPD706
CMNPD10257	CMNPD15857	CMNPD23645	CMNPD3215	CMNPD708
CMNPD104	CMNPD16757	CMNPD23677	CMNPD3219	CMNPD709
CMNPD105	CMNPD16761	CMNPD23678	CMNPD3220	CMNPD71
CMNPD112	CMNPD16766	CMNPD23679	CMNPD3222	CMNPD718
CMNPD113	CMNPD16767	CMNPD24296	CMNPD3225	CMNPD727
CMNPD114	CMNPD16770	CMNPD24297	CMNPD3226	CMNPD7340
CMNPD115	CMNPD17492	CMNPD24301	CMNPD357	CMNPD736
CMNPD11748	CMNPD17525	CMNPD24306	CMNPD359	CMNPD742
CMNPD11772	CMNPD17821	CMNPD24307	CMNPD3638	CMNPD743
CMNPD11773	CMNPD17823	CMNPD24308	CMNPD3639	CMNPD747
CMNPD11778	CMNPD1850	CMNPD25039	CMNPD3687	CMNPD748
CMNPD11787	CMNPD1868	CMNPD25040	CMNPD3710	CMNPD749
CMNPD11791	CMNPD1869	CMNPD25041	CMNPD3715	CMNPD750
CMNPD11810	CMNPD1871	CMNPD25042	CMNPD3718	CMNPD758
CMNPD125	CMNPD1872	CMNPD25044	CMNPD3721	CMNPD759
CMNPD12500	CMNPD1873	CMNPD25047	CMNPD3724	CMNPD761
CMNPD12501	CMNPD1880	CMNPD25050	CMNPD3727	CMNPD763
CMNPD12503	CMNPD1883	CMNPD25701	CMNPD4006	CMNPD773
CMNPD12535	CMNPD1887	CMNPD2630	CMNPD4206	CMNPD775
CMNPD12536	CMNPD1888	CMNPD2641	CMNPD4208	CMNPD793
CMNPD12540	CMNPD1891	CMNPD27069	CMNPD4210	CMNPD794
CMNPD12543	CMNPD18959	CMNPD27080	CMNPD4219	CMNPD8069
CMNPD12544	CMNPD18962	CMNPD2775	CMNPD4777	CMNPD859
CMNPD12545	CMNPD18963	CMNPD27809	CMNPD4783	CMNPD86
CMNPD12548	CMNPD18964	CMNPD27848	CMNPD4786	CMNPD8853
CMNPD12553	CMNPD18972	CMNPD27856	CMNPD4788	CMNPD8855
CMNPD12554	CMNPD18978	CMNPD27859	CMNPD4789	CMNPD8860
CMNPD12555	CMNPD18982	CMNPD28398	CMNPD4791	CMNPD90
CMNPD12556	CMNPD1899	CMNPD29253	CMNPD5337	CMNPD91
CMNPD12557	CMNPD1904	CMNPD29266	CMNPD5392	CMNPD94
CMNPD12558	CMNPD1911	CMNPD29281	CMNPD5393	CMNPD95
CMNPD13353	CMNPD19957	CMNPD29294	CMNPD5893	CMNPD9527
CMNPD13355	CMNPD19959	CMNPD29295	CMNPD5938	CMNPD97
CMNPD13357	CMNPD19960	CMNPD29302	CMNPD5966	CMNPD99
CMNPD13358	CMNPD19961	CMNPD29313	CMNPD61	
CMNPD13362	CMNPD19997	CMNPD29314	CMNPD661	
CMNPD13364	CMNPD20002	CMNPD29315	CMNPD6631	
CMNPD13366	CMNPD20003	CMNPD29317	CMNPD6636	
CMNPD14898	CMNPD20633	CMNPD31040	CMNPD6640	
CMNPD14904	CMNPD21126	CMNPD31051	CMNPD6643	
CMNPD14905	CMNPD21145	CMNPD31053	CMNPD6644	
CMNPD14910	CMNPD22367	CMNPD31512	CMNPD6648	
CMNPD14915	CMNPD22369	CMNPD31513	CMNPD665	

**Table 2:** Structure validation results of different servers for the generated homology model of 3-oxoacyl synthase II of *N. meningitidis.*

**Server Name**	**Result**
PROCHECK	Residues present inthe most favoured regions = 90.5%
	Residues present in the disallowed regions = 0.6%
ERRAT	Overall Quality Factor = 90.0383
VERIFY3D	Pass
ProSA-web	Z-Score = -9.14

**Table 3:** Binding energies of 3 selected marine phytochemicals and their plant source showing better binding affinity than standard (Minocycline) when docked against 3-oxoacyl synthase II of *N. meningitidis.*

**S.No.**	**Ligand**	**Binding energy (kCal/mol)**	**Plant species**
1	Minocycline	-7.46	---
2	CMNPD5392	-8.95	*Osmundeahybrida*
3	CMNPD14910	-8.14	*Laurenciaokamurae*
4	CMNPD14926	-7.98	*Laurencia sp.*
